# Unexpected structure for the N-terminal domain of hepatitis C virus envelope glycoprotein E1

**DOI:** 10.1038/ncomms5874

**Published:** 2014-09-16

**Authors:** Kamel El Omari, Oleg Iourin, Jan Kadlec, Geoff Sutton, Karl Harlos, Jonathan M. Grimes, David I. Stuart

**Affiliations:** 1 Division of Structural Biology, The Wellcome Trust Centre for Human Genetics, University of Oxford, Headington, Oxford OX3 7BN, UK; 2 Diamond Light Source Limited, Harwell Science and Innovation Campus, Didcot OX11 0DE, UK; 3 These authors contributed equally to this work; 4 Present address: EMBL Grenoble, 71 avenue des Martyrs, 38042 Grenoble, France

## Abstract

Hepatitis C virus (HCV) infection remains a major health problem worldwide. HCV entry into host cells and membrane fusion are achieved by two envelope glycoproteins, E1 and E2. We report here the 3.5-Å resolution crystal structure of the N-terminal domain of the HCV E1 ectodomain, which reveals a complex network of covalently linked intertwined homodimers that do not harbour the expected truncated class II fusion protein fold.

## Abstract (web summary)

Hepatitis C virus (HCV) gains entry into host cells via envelope glycoproteins E1 and E2. Here, El Omari *et al.* present the crystal structure of the N terminus of the E1 ectodomain of HCV and show that it adopts a different fold than predicted.

## Graphical abstract

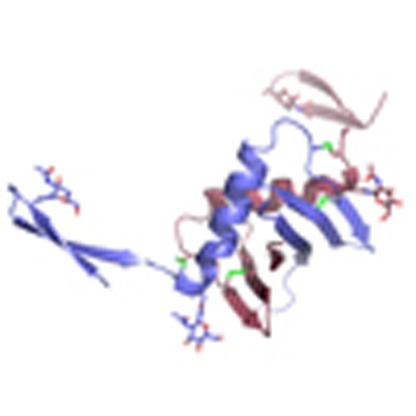

Hepatitis C virus (HCV) is a major human pathogen responsible for chronic liver diseases such as hepatocellular carcinoma and cirrhosis. Between 130 and 170 million people are probably chronically infected^1^, and HCV-related diseases cause the death of some 350,000 people every year^2^. New HCV drugs have been approved (telaprevir and boceprevir) but research is still ongoing because of the high cost of these drugs^3^.

HCV is a small positive-sense single-stranded RNA-enveloped virus belonging to the hepacivirus genus of the *Flaviviridae* family. Its genome encodes a single polyprotein processed by proteases into 10 proteins. Of the three structural proteins, E1, E2 and Core, the envelope glycoproteins E1 and E2 are responsible for cell attachment and viral fusion. Both are type I transmembrane proteins, with highly glycosylated N-terminal ectodomains containing 8 and 18 conserved cysteines, respectively^4^. Within cells E1 and E2 form non-covalent heterodimers, and on the virion, high-molecular mass complexes stabilized by disulphide bonds^5^. E2 and E1 mediate interactions with host proteins such as CD81 (ref. 6) and alipoprotein E (ApoE) respectively^7^.

Recently, the crystal structures of *Flaviviridae* E2s from bovine viral diarrhoea virus^8^ and HCV^9^,^10^ have revealed folds that were unexpectedly different^11^ from class II fusion proteins, and from each other, however, no structural information on E1 glycoproteins is available. Hence we have determined the crystal structure of the N-terminal domain (residues 1–79) of HCV glycoprotein E1 ectodomain (nE1) at 3.5 Å resolution by multi-crystal single-wavelength sulphur anomalous dispersion providing the first structural insights into a *Flaviviridae* E1 glycoprotein^12^, revealing a covalently linked dimer with an unexpected fold.

## Results

### Overall fold

**Figure 1:**
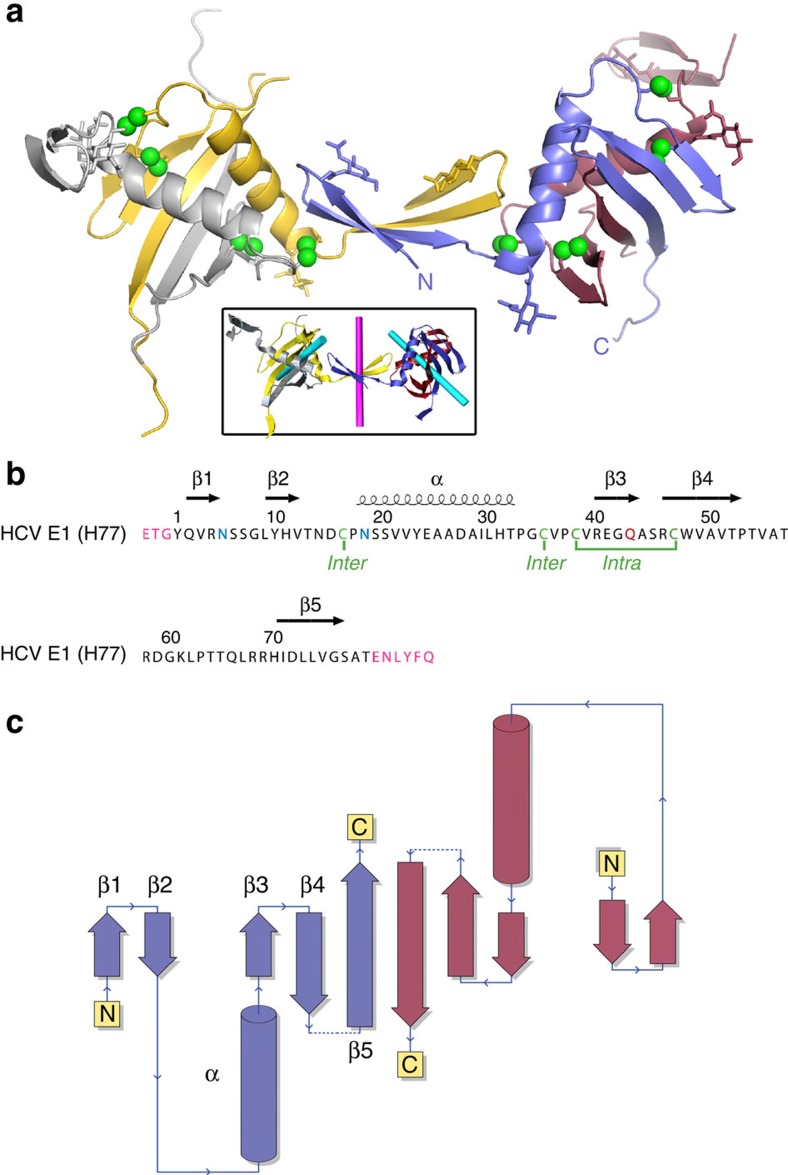
Overall fold of HCV nE1. (**a**) Cartoon representation of a dimer of dimers. Each monomer is coloured differently. Sulphur atoms forming disulphide bridges are shown as green spheres and sugars as sticks. The box shows the twofold axis at covalent and non-covalent dimer interfaces in cyan and magenta, respectively. (**b**) The amino-acid sequence of nE1 has been aligned with its secondary structure elements with ESPRIPT^32^. Cysteines involved in disulphide bonds (intra- and inter-chains) are shown in green, glycosylation sites in blue and the N43Q mutation in red. Extra residues added by the cloning procedures are shown in pink; the ENLYFQ sequence corresponds to the TEV cleavage site. (**c**) Topology of a covalently linked dimer of nE1, coloured identically to **a**.

nE1 consists of a β-hairpin (residues 1–14) followed by a domain composed of a 16 amino-acid long α-helix (residues 17–32) flanking a three-strand antiparallel β-sheet (Fig. 1). The loop between strands β4 and β5 contains 10 disordered residues that are not seen in electron density. We do not exclude the possibility that the disordered residues can be involved in domain swap, but we think it is rather unlikely.

### Dimer interfaces

**Figure 2:**
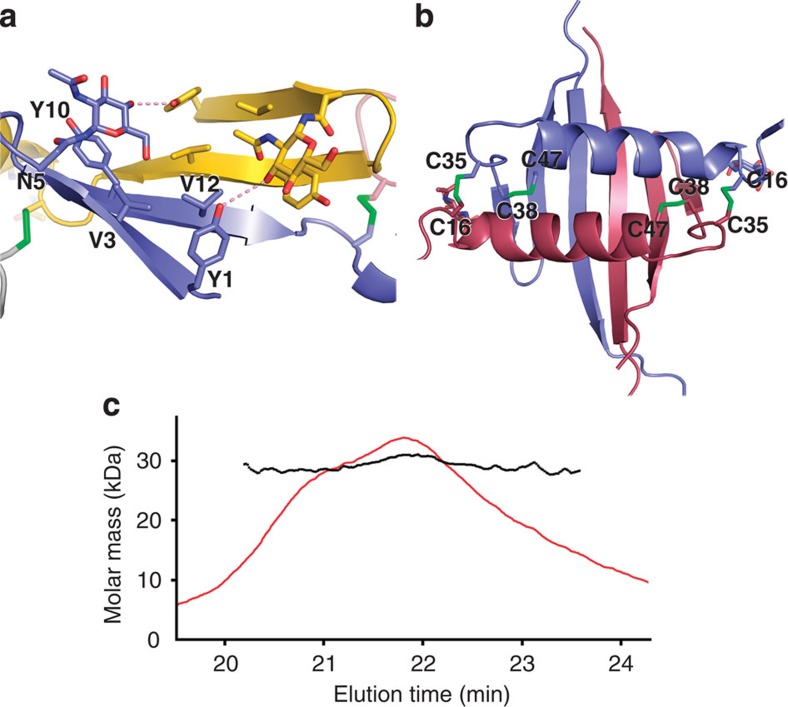
HCV nE1 oligomerization. (**a**,**b**) Cartoon and sticks representation of the non-covalent (**a**, blue and yellow monomers) and covalent (**b**, blue and red monomers) interfaces. Chains are coloured as in (Fig. 1a). Nitrogen, oxygen and sulphur atoms are coloured blue, red and green, respectively. Pink dashes show hydrogen bonds between tyrosines 1 and sugars. (**c**) Multi-angle light-scattering profile showing that HCV nE1 forms dimers in solution. The theoretical mass expected for a glycosylated dimer would be around 28.8 kDa. Two peaks are present (at 21 and 22 min elution time) with corresponding molecular masses of 28.5 and 30.3 kDa; both molecular weights have a polydispersity of 1. The two peaks probably represent different glycosylation states.

**Figure 3:**
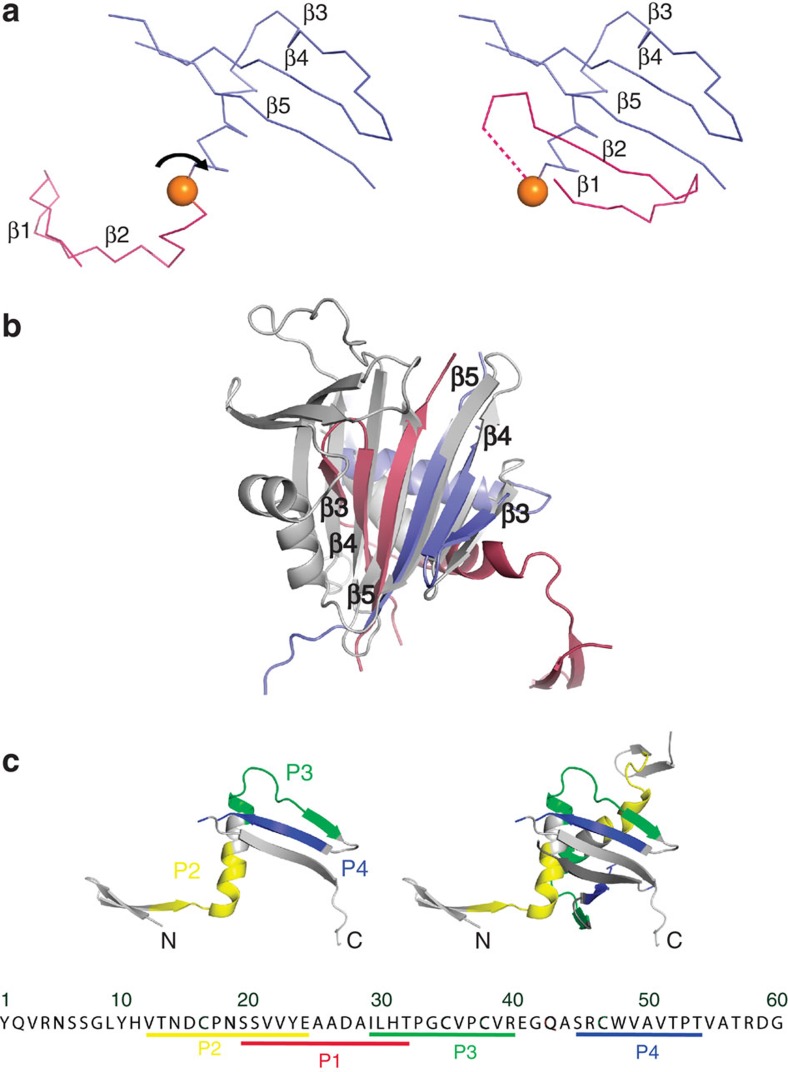
Structural comparison and interaction of nE1. (**a**) Suggested rearrangement of the β-hairpin in HCV E1 monomers. On the left, ribbon representation of a nE1 monomer found in the crystal structure. In monomeric or heterodimeric conformations of HCV E1, the β-hairpin (pink) could fold back to extend the three-stranded β-sheet coloured in blue (one possible conformation is shown on the right). (**b**) Superposition of HCV nE1 covalent dimer (red and blue) on human phosphatidylcholine transfer protein (grey). Structures are drawn in cartoon representation. (**c**) HCV E1 peptides P1, P2, P3 and P4 shown to bind apolipoproteins coloured red, yellow, green and blue, respectively, mapped onto nE1 monomer (top left), dimer ( top right) and the sequence of the first 60 residues of HCV nE1 (bottom). For clarity, peptide P1, which overlaps onto P2 and P3, is not shown.

There are six monomers in the crystallographic asymmetric unit, forming two types of dimer interfaces and a protein network stabilized by the flexibility of the hinge region around residue S20 between the β-hairpin and the adjacent domain^13^ (Supplementary Fig. 1). The first interface (700 Å^2^) is formed by the interaction of the N-terminal β-hairpins forming an antiparallel β-sheet, and by hydrogen bonding between *N*-acetyl-D-glucosamine linked to N5 and the Y1 hydroxyl group (Fig. 2a). The second interface is much more extensive (1,400 Å^2^), resulting from the interaction of two β5 strands to form a six-strand β-sheet on one face of which the helices of the two monomers pack side-by-side (Fig. 2b). This interface is covalently braced by two intermolecular disulphide bridges between residues C16 and C35. The two remaining cysteines, C38 and C47, form an intramolecular covalent bond between strands β3 and β4 (Figs 1b and 2b). The larger surface area and rather hydrophobic nature of the buried surfaces together with the precise constraints placed on this interaction by the disulphide bonds strongly suggest that this is a biologically relevant interaction, whereas the smaller interface has very few specific interactions involving side chains. This hypothesis is confirmed by the fact that nE1 forms covalent dimers in solution, as shown by multi-angle light-scattering experiments (Fig. 2c) and non-reducing SDS–polyacrylamide gel electrophoresis^12^. However, both interfaces have a similar shape complementarity (SC=0.76)^14^, and it is possible that the β-hairpin could fold back in monomeric E1 to replace the dimer interface (Fig. 3a). This hypothesis suggests that the switch from heterodimeric to homodimeric E1 could be concomitant with a domain swap.

## Discussion

Supporting the biological relevance of the disulphide-stabilized dimer interface, it has been proposed that the presence of disulphide bonds improves lateral interactions between glycoproteins on the virion and might play a role in the budding of HCV particles^5^,^15^. E1 homodimers might be built into a higher-order complex through cysteines of E2 and the C-terminal domain of E1. A different study suggested that E1 and E2 contain reduced cysteines crucial for viral entry, which would form disulphide bonds following interaction of HCV with cell-surface receptors^16^. The reduced cysteines have not been identified; it is also possible that the covalent nE1 dimer might correspond to the post-attachment conformation.

Bioinformatic studies suggested that HCV E1 has a truncated class II fusion fold^17^, whereas the structural data reported here demonstrate that nE1 does not resemble this fold. In fact the architectures of HCV E1, E2 and bovine viral diarrhoea virus E2 are not comparable to any class I, II or III fusion proteins. This suggests that there may be quite different fusion mechanisms employed within the family *Flaviviridae*, with HCV differing from the pestiviruses and both differing from the better-understood mechanism employed by alphaviruses and flaviviruses. Furthermore, HCV glycoproteins are rather small compared with known fusion proteins, and E2 has a compact globular fold^9^, only half as long as expected for a viral protein that reaches the host membrane. In addition, the E1E2 heterodimer is necessary for viral fusion, unlike alphaviruses or flaviviruses where E1 or E trimers, respectively, are sufficient. It seems inescapable that a novel fusion mechanism should be considered for HCV.

A DALI search^18^ using the nE1 dimer reveals that the closest structural homologue is a phosphatidylcholine transfer protein (PDB code 1LN2, *z*=4.9, r.m.s.d.=4.2 Å, number of matches=74); the extended β-sheet matches in part the steroidogenic acute regulatory protein-related transfer domain responsible for binding hydrophobic ligands, such as sterols and lipid-like molecules (Fig. 3b). In nE1, the two β5-strands interact with the hydrophobic leucine and phenylalanine of the C-terminal tobacco etch virus (TEV) protease site (Supplementary Fig. 2), which in our construct replaces the start of a hydrophobic putative fusion peptide^19^,^20^,^21^. Even if this packing is adventitious, the rather hydrophobic surface that the TEV site packs against is likely to be involved in interaction with another protein element. Indeed the N-terminal domain of HCV E1 has been suggested to be responsible for the binding to ApoB and ApoE and facilitates virus entry through either low-density lipoprotein receptor^7^ or heparin sulphate^22^. Our structure contains the HCV E1 peptides (P1–P4) that supposedly interact with ApoE^7^ (Fig. 3c), suggesting that nE1 is exposed on the surface of the virion.

Effectively developing anti-HCV drugs and vaccines requires a detailed knowledge of the mechanism of crucial viral events such as membrane fusion, and recent structural results (for HCV E2 core, and nE1 presented here) indicate that the accepted class II fusion model for HCV is incorrect and provide a start point for understanding what will likely be a novel fusion machinery.

## Methods

### Cloning

Codon-optimized DNA encoding the first 79 residues of the envelope glycoprotein E1 of the prototypic strain H77 (corresponding to residues 192–270 of the polyprotein, UniProt B0FYC5) fused at its C terminus to a TEV protease cleavage site (ENLYFQ) was synthesized (Life technologies). The synthetic gene was designed with a mutation N43Q to remove this N-linked glycosylation site. The complementary DNA was cloned into the pHLsec vector^23^ between the AgeI and KpnI restriction sites, resulting in secreted protein with a C-terminal His_6_-tag. The final sequence and the mutation were verified by DNA sequencing.

### Protein expression and purification

The N43Q mutant construct was transiently expressed in HEK 293T cells in the presence of 5 μM of the *N*-glycosylation inhibitor kifunensine (Toronto Research Chemicals, North York, ON, Canada). The cell media, containing the secreted HCV E1, was harvested 4 days after transfection after centrifugation at 5,000 *g* at 15 °C for 20 min to remove debris. The media was filtered and incubated with Ni^2+^-charged resin (FF Chelating Sepharose resin, GE Healthcare) in a shaking incubator for 90 min at 15 °C. The resin was separated from the media, washed with 15 mM Tris-HCl pH 8.0, 0.1 M NaCl, 20 mM imidazole and the bound protein was eluted with the same buffer but containing 250 mM imidazole. To remove the C-terminal His-tag and to deglycosylate the protein, the eluted protein was incubated overnight at 22 °C with recombinant TEV protease and endoglycosidase F1 produced inhouse. The supernatant was filtered and the protein purified by size-exclusion chromatography in 20 mM Tris-HCl pH 7.0 and 0.1 M NaCl on a Superdex 75 column (GE Healthcare). Fractions containing pure protein (nE1) were collected and 3-(1-Pyridino)-1-propane sulphonate (NDSB 201, Soltec Ventures Inc.) was added to a final concentration of 300 mM, to enable the protein to be concentrated to between 17 and 22 mg ml^−1^.

### MALS

Multi-angle light-scattering (MALS) experiments were carried out using an analytical Superdex S200 10/30 column (GE Heathcare) with online static light scattering (DAWN HELEOS II, Wyatt Technology, Santa Barbara, CA), differential refractive index (Optilab rEX, Wyatt Technology) and Agilent 1200 UV (Agilent Technologies) detectors. Protein used for MALS had previously been purified as described above, but not deglycosylated, and concentrated to ~4.2 mg ml^−1^. Data were analysed using the ASTRA software package (Wyatt Technology).

### Crystallization and data collection

Crystals of nE1 were grown by sitting-drop vapour diffusion at 20.5 °C, in 96-well plates (Greiner Bio-One Ltd, Stonehouse, UK). Plates were set up with a Cartesian Technologies MIC4000 robot, as previously described^24^.

nE1 crystallized in 15% (wt/v) polyethylene glycol 1500, 3.6% (wt/v) polyethylene glycol 4000 and 0.05 M sodium acetate pH 4.8. Crystals were of either hexagonal or tetragonal morphology of which the latter gave better diffraction. Addition of 100 nl of 6–8% of 2,5-hexanediol or 1,6-hexanediol lead to larger crystals of approximate size 110 × 30 × 10 μm.

Crystals were flash frozen into liquid nitrogen using 25% (v/v) ethylene glycol/resevoir solution as cryoprotectant. Although the crystals diffracted very weakly, data sets could be recorded from crystals at 100 K at Diamond Light Source (Didcot, UK, beamlines I24 and I04) and were processed with either HKL2000 (ref. 25) or XIA2 (ref. 26) in the space group *P*4_1_2_1_2 with unit cell dimensions *a*=*b*=105.0 Å, *c*=204.8 and *α*=*β*=*γ*=90°. For the native data collection, numerous crystals were screened to eventually get a crystal diffracting to higher resolution (3.5 Å), whereas for the sulphur single-wavelength anomalous dispersion (Sulphur-SAD) data collection 32 crystals were used. Processing statistics are summarized in Table 1.

### Structure determination and refinement

Experimental phasing with heavy atoms was unsuccessful and the absence of methionines prevented the use of selenomethionine for structure determination. Nonetheless, HCV E1 (residues 1–79) contains four cysteines, thus data sets from 32 crystals were collected at a wavelength of 1.77 Å at beamline I04 using inverse beam mode in 5° wedges and merged with XIA2 (Table 1) to use sulphur-SAD phasing. HKL2MAP was used to locate sulphur sites using the merged data cut to 7 Å resolution (if cysteines are involved in disulphides, the anomalous signal decays rapidly beyond this point)^27^. Twelve sulphur sites (presumed to be the centroids of disulphide bonds) were input to PHENIX autosol^28^ giving electron density maps that were difficult to interpret; however, it was possible to place six α-helices in the map from which non-crystallographic symmetry (NCS) matrices could be extracted. Density modification using a solvent content of 75%, sixfold NCS averaging and extension of the resolution to the native data set (3.5 Å) gave interpretable maps. The location of the cysteines and glycans allowed the alignment of the sequence into density. Model building was performed with COOT^29^ and refinement with AUTOBUSTER, using sixfold NCS restraints automatically generated by the local structural symmetry restraints^30^ (Supplementary Fig. 1c) The final *R*_work_/*R*_free_ values of the model were 22/24% and geometry was checked with MolProbity^31^. For refinement statistics see Table 1. Data collection and structure determination are described in greater details elsewhere^12^.

## Author contributions

K.E.O. collected data, solved and refined the structure. O.I. and J.K. carried out cloning, purification and crystallization. G.S. performed MALS experiments. K.H. performed crystal mounting and collected data. K.E.O., J.M.G. and D.I.S. analysed the structures and wrote the manuscript. D.I.S. supervised the project.

## Additional information

**Accession codes:** Coordinates and structure factors of HCV nE1 have been deposited in the Protein Data Bank under ID code 4UOI.

**How to cite this article:** El Omari, K. *et al.* Unexpected structure for the N-terminal domain of Hepatitis C virus envelope glycoprotein E1. *Nat. Commun.* 5:4874 doi: 10.1038/ncomms5874 (2014).

## Supplementary Material

Supplementary Information Supplementary Figures 1-2

## Figures and Tables

**Table 1:** Data collection and refinement statistics.

	**Sulphur-SAD**	**Native**
*Data collection*		
Space group	*P*4_1_2_1_2	
Cell dimensions		
*a*, *b*, *c* (Å)	105.5, 105.5, 204.8	105.0, 105.0, 204.7
*α*, *β*, *γ* (°)	90, 90, 90	90, 90, 90
Resolution (Å)	60.3–4.2 (4.32–4.21)	50–3.5 (3.63–3.50)
*R*_merge_	16.0 (35.2)	13.5 (81.0)
*I*/*σI*	33.3 (3.6)	17.6 (2.2)
Completeness (%)	99.4 (96.3)	99.5 (99.5)
Redundancy	121.5 (4.2)	6.2 (6.2)
		
*Refinement*		
Resolution (Å)		31.3–3.5
No. of reflections		15,137 (1,471)
*R*_work_/*R*_free_		0.216/0.237
No. of atoms		
Protein		3,071
Ligand		140
B-factors (Å^2^)		
Protein		116.4
Ligand		140.1
R.m.s.d.		
Bond lengths (Å)		0.008
Bond angles (°)		1.13
