# Supplementary material for: Unexpected structure for the N-terminal domain of hepatitis C virus envelope glycoprotein E1
Source: Nat Commun. 2014 Sep 16;5:4874. doi: 10.1038/ncomms5874 (PMC4175578; doi:10.1038/ncomms5874)
Supplement: Supplementary Information — Supplementary Figures 1-2 [file ncomms5874-s1.pdf]

Supplementary Figures

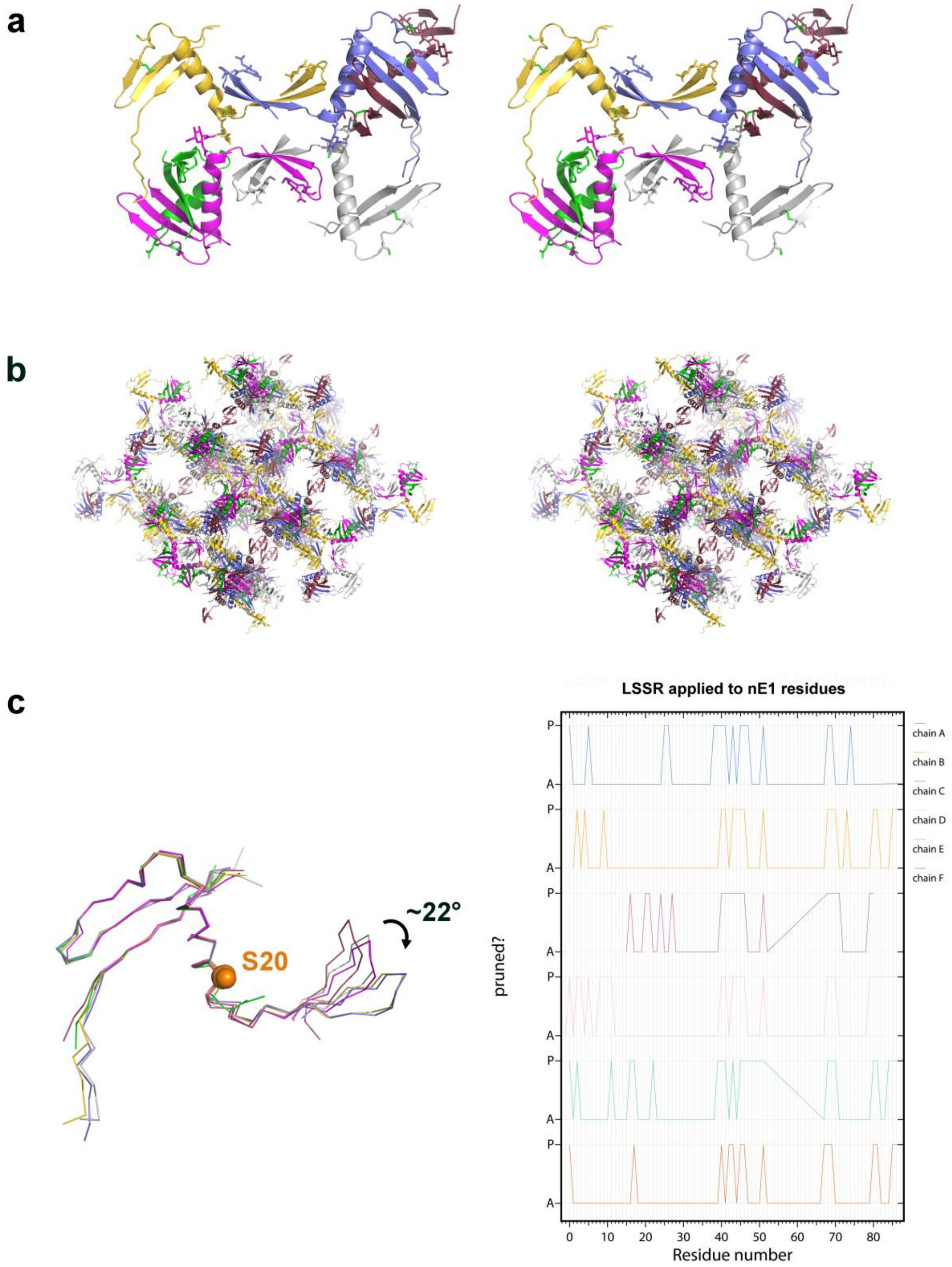

Supplementary Figure 1: Crystal packing and flexibility of HCV nE1

(a) Stereo view of the six monomers forming the asymmetric unit, each monomer of the asymmetric unit is colored differently. **(b)** Stereo view of the network of interaction of HCV nE1 within the crystal. Monomers are colored as in (a). **(c)** On the left, different conformations of HCV nE1 β-

hairpins observed in the six monomers present in the asymmetric unit. Monomers superposed on the domain harboring the α-helix, are shown in ribbon representation and colored as in (a). The orange spheres represent the hinge residue S20. On the right, plot showing the residues of each monomer, active residues (A) are restrained by AUTOBUSTER using local NCS restraints, pruned residues (P) are those which have been automatically removed from local structural symmetry restraints.

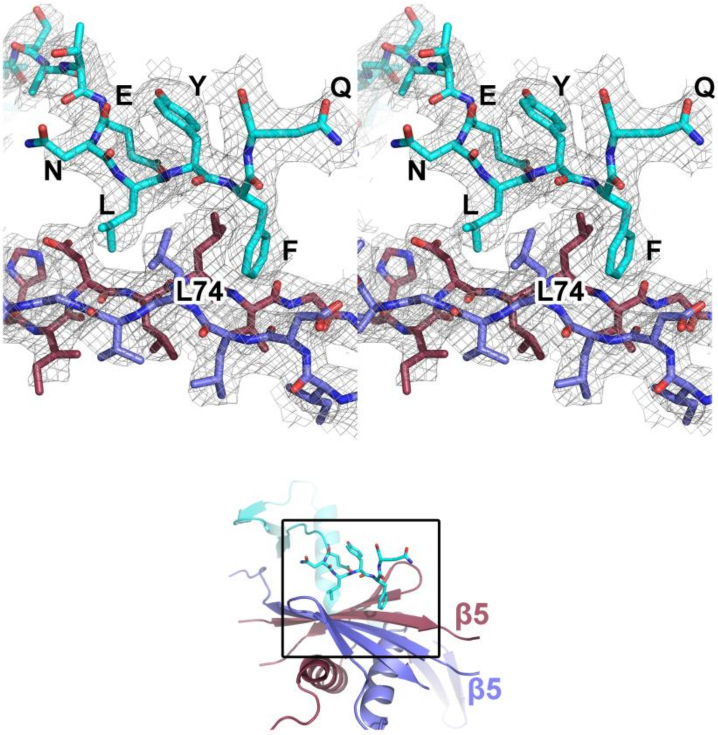

Supplementary Figure 2: Putative interaction site of HCV nE1 dimer

Interaction of the TEV protease site from an adjacent monomer (cyan) with a HCV nE1 covalent dimer (red and blue). The grey mesh shows the 2Fo-Fc electron density contoured at 1σ.
